# Secure Edge-Based Energy Management Protocol in Smart Grid Environments with Correlation Analysis

**DOI:** 10.3390/s22239236

**Published:** 2022-11-27

**Authors:** Amjad Rehman, Khalid Haseeb, Gwanggil Jeon, Saeed Ali Bahaj

**Affiliations:** 1 Artificial Intelligence and Data Analytics (AIDA) Lab, CCIS Prince Sultan University, Riyadh 11586, Saudi Arabia; 2 Department of Computer Science, Islamia College Peshawar, Peshawar 25120, Pakistan; 3 Department of Embedded Systems Engineering, Incheon National University, Incheon 22012, Republic of Korea; 4 MIS Department, College of Business Administration, Prince Sattam Bin Abdulaziz University, Alkharj 11942, Saudi Arabia

**Keywords:** smart grid, machine learning, communication privacy, conservation, cloud storage

## Abstract

For the monitoring and processing of network data, wireless systems are widely used in many industrial applications. With the assistance of wireless sensor networks (WSNs) and the Internet of Things (IoT), smart grids are being explored in many distributed communication systems. They collect data from the surrounding environment and transmit it with the support of a multi-hop system. However, there is still a significant research gap in energy management for IoT devices and smart sensors. Many solutions have been proposed by researchers to cope with efficient routing schemes in smart grid applications. But, reducing energy holes and offering intelligent decisions for forwarding data are remain major problems. Moreover, the management of network traffic on grid nodes while balancing the communication overhead on the routing paths is an also demanding challenge. In this research work, we propose a secure edge-based energy management protocol for a smart grid environment with the support of multi-route management. It strengthens the ability to predict the data forwarding process and improves the management of IoT devices by utilizing a technique of correlation analysis. Moreover, the proposed protocol increases the system’s reliability and achieves security goals by employing lightweight authentication with sink coordination. To demonstrate the superiority of our proposed protocol over the chosen existing work, extensive experiments were performed on various network parameters.

## 1. Introduction

The IoT can connect people and things with anyone and anything at any time, anywhere, and over any network with the support of wireless technologies [1,2,3]. It is a vast, dynamic global network infrastructure comprising web-enabled, Internet-connected objects [4,5,6]. Smart grids are one of the most significant IoT applications that collect data from transmission lines, distribution substations, and consumers. The collected data are forwarded to cloud storage for processing and analysis. A smart grid needs endless, reliable, and real-time connectivity because it handles a significant amount of data and standards [7,8,9]. Smart meters generate a lot of data, and it might be difficult to store, process, and analyze the data because they vary in size and velocity. Thus, cloud computing is typically used to store and analyze the data produced by a smart grid since it offers real-time response with scalability for a variety of systems [10,11]. Smart technologies are interconnected with the support of sensors to sense the surrounding data and transmit them in a multi-hop manner toward the sink node [12,13,14].

Although numerous methods have been developed for the effective management of data routing in smart grid applications [15,16,17], selecting the optimal multipath while including learning techniques for the autonomous system is a crucial responsibility [18,19,20]. The learning approaches with the integration of smart sensors have received much research interest. These methods enable autonomous systems to gather and understand the huge amount of data from remote devices [21,22,23]. Although IoT and wireless systems offer a variety of capabilities for smart grids, their combination causes many research hurdles for communication [24,25,26]. Such a communication system leads to compromising the sensitive data of a smart grid and decreases device-level trust. Therefore, the purpose of this work is to propose and develop a secure edge-based sensing protocol that efficiently uses energy resources and distributes collected data promptly. The proposed protocol uses numerous paths and trains the system to achieve sustainable communication by predicting the node’s behavior based on correlation analysis. Moreover, cryptographic approaches are capable of detecting malicious activities on grid data and defending the system from threats.

The following highlight the primary contributions of the proposed protocol.

- A minimal cost value for smart devices is explored for the development and management of energy-efficient smart grids.
- A correlation technique is utilized for forwarding tables and extracting the optimal choices for the prediction of routing paths.
- To provide authorized access, the edge network and sink node collaborate securely to promptly communicate the sensed data.
- The proposed protocol is validated using extensive simulations and experimental results are discussed.

The remaining paper is organized as follows. Section 2 discusses the related work and highlights existing problems. Section 3 demonstrates the significance of the proposed protocol. Section 4 discusses the details of the proposed protocol with the network model. Section 5 explains the system configuration and experimental results. In Section 6, the research work is concluded.

## 2. Literature Review

Smart grid systems are expanding and developing due to the integration of IoT networks. Moreover, wireless technologies facilitate the grid environment obtaining environmental behaviors and conditions [27,28,29]. Such technologies manage the information collection and flow of data toward cloud systems with the support of edge computing. For smart grid WSNs, the authors [30] proposed an energy-efficient adaptive fuzzy-based multidisjoint routing protocol (MDRP). The protocol offers an energy-efficient way of choosing the next hop by exploring fuzzy logic. The sink node is used as the root of a spanning tree that is constructed when the next node is chosen to compute the optimal path cost for data transferring. Moreover, the proposed MDRP offers improved performance in terms of network lifetime, packet delivery ratio, and energy utilization. The authors in [31] proposed a unique dynamic-clustering-based, energy-efficient, and quality-of-service (QoS)-aware routing protocol (EQRP) that is motivated by the actual behavior of bird mating optimization (BMO). For WSN-based smart grid applications, the proposed distributed method considerably raises network dependability and decreases unnecessary packet retransmissions. Performance data demonstrated that the proposed protocol successfully decreased end-to-end latency and improved memory utilization, packet delivery ratio, residual energy, and throughput.

In [32], the authors provided an energy-efficient grid-based routing method for sensor networks to maintain the energy in sensor nodes and lengthen the lifetime of the network. To reduce the number of hops in the routing process, routing is also carried out by a grid coordinator that employs fuzzy rules to select the best route. The simulations performed for that work showed that the suggested routing algorithm outperformed other grid and cluster-based routing protocols in terms of residual energy and network lifetime. The authors in [33] proposed a real-time monitoring edge computing system and shift processing from a centralized cloud to edge servers that are close to the device. They developed a scheduling problem to further improve the framework to maximize the benefits and provided an intelligent heuristic approach based on the simulated annealing technique. The experiments showed that the proposed framework could enhance the monitoring frame rate by up to 10 times, and decrease the detection delay by up to 85% as compared to the cloud monitoring solution. In [34], preprocessing, clustering, feature selection, and classification were combined to improve security and learning practices. Initially, the network data were clustered using the Markov chain clustering (MCC) model after the feature values had been normalized. The rapid probabilistic correlated optimization (RPCO) process was then used to determine the matching score and particle likelihood in order to choose the optimal features. The predicted label was then classified using the block-correlated neural network (BCNN) method, where the relevancy score is determined by combining feature points and the kernel function. Different performance indicators were employed in the experiments to verify the outcomes of the proposed techniques.

To collect the sensed data by these cell nodes, the authors in [35] proposed a mechanism for geographically segmenting the network into several cells. Cells were split into two groups based on how they communicated with mobile sinks: single-hop communication cells (SCCs) and multi-hop communication cells (MCCs). Mobile sinks moved over two concentric diamond-shaped orbits, such that one sink was always covering one-half of the network. To collect data from sensor nodes, both sinks initially traveled in one direction and stopped at specific intervals in the corners of the orbits. SCCs delivered data directly to sinks when they are stationary, but MCCs used the proposed routing algorithm (EGRPM) to send data to mobile sinks. In [36], the authors provided an energy-aware cluster-based routing protocol (EACRP) for CRSN that simultaneously took into account both energy and dynamic spectrum problems. The reported CRSN schemes are energy-inefficient and have high re-clustering frequency caused by PU operations. The proposed method used self-organized distributed clustering to create an ideal number of clusters, resulting in reduced average node power. The proposed method created clusters with a greater number of common channels to lessen the impact of PU activity and used the idea of cooperative sensing to choose the data channels for intra-cluster communication. EACRP chooses gateway nodes with higher energy, closer proximity to the sink node, and a greater number of shared channels with nearby nodes to improve inter-cluster connectivity.

## 3. Significance of the Proposed Protocol

IoT devices are growing in the collection, processing, and forwarding of the data of smart grids. Such data must be delivered on time to cloud storage for additional data analysis and to quickly respond to distributed applications. Smart devices not only collect environmental data, but they must also make decisions intelligently before transferring environmental data to cloud systems. Recently, many schemes have been presented for coping with routing issues by reducing energy consumption for smart sensors. However, the majority of existing solutions are unable to effectively control traffic on multiple paths, and fail to maintain the least amount of data damage. Moreover, very few solutions can secure the smart grid, and the sensor network is vulnerable as a result of the presence of multifunctional devices. The proposed protocol examines machine-learning strategies for routing sensing data toward the sink node with the support of gateway devices. The edges perform the role of gateway services and facilitate communication for both the lower and upper tiers. Furthermore, with the support of cryptography principles, the proposed protocol protects the data over unreliable networks in terms of security objectives.

## 4. Proposed Secure Edge-Sink Collaborated Energy Management Protocol

This section presents a detailed discussion of the proposed protocol, developed for crucial wireless applications in a smart grid environment. To meet the requirements of a smart system with the incorporation of energy efficiency, reliable communication, and secure data management, the proposed protocol provides the least-costly solution with the collaboration of machine-learning techniques and securing sensing data against illegal events. It also provides optimal multipath routes for the transmission of sensor data at a manageable cost.

### 4.1. System Assumption and Network Model

In the proposed protocol, each node including sink and edge devices has unique identities assigned. The network field comprises various sensors that are connected in the form of a grid to transmit the data to the sink node. Each sensor node has homogeneous resources with a fixed transmission radius R. The following are the system assumptions before designing and explaining the proposed protocol.

- Smart sensors have minimal transmission range and mobility features.
- IoT sensors have a preinstalled global positioning system (GPS).
- The sink node is static and all the nodes are deployed randomly.
- Each node has enough memory to store and maintain its forwarding tables.
- Edge devices are placed between the IoT and sink node.

**Figure 1:**
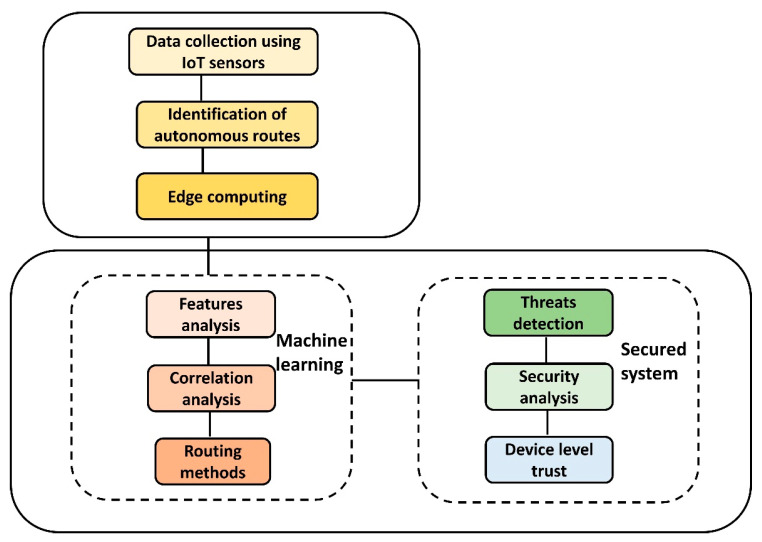
Block diagram of the proposed protocol.

The design of the proposed protocol was composed of two phases. In the first phase, smart sensors were arranged in a unidirectional graph G. There were separate links, also called edges E, between consecutive nodes N. At the start, a distinct value is assigned to each link using the distance parameter; accordingly, each node maintains its forwarding table. The routing is achieved in the form of a multi-hop to reduce the power consumption of the grid nodes. In the proposed protocol, the sink node is assumed to be stronger than the devices due to its adequate processing, storage, transmission, and energy resources. Each node maintains its local information and updates its neighbor to avoid problems in routing decisions. The block diagram for the proposed protocol is shown in Figure 1. It consists of three main phases. First, data are collected from the grid system using the sensors of IoT devices with the integration of wireless algorithms. With the support of edge devices, the data are verified and border nodes are authenticated. Second, using correlation analysis, the weighted value is determined by exploring certain factors. Such a factor balances the load and energy conversation among the grid nodes, and also prolongs the network lifetime. In the end, with the support of the sink node, a strong and more reliable security algorithm is developed that authenticates the devices using hybrid keys and performs integrity functions to attain the trust of the device.

### 4.2. Discussion

This section explains the proposed protocol. It comprises two components to cope with transmissions and securing data. After node initialization in the grid system, each node creates and stores the collected information inside its forwarding table. The format of the forwarding table consists of the node’s identity ID, distance d, mobility coordinates (x,y), and value of neighborhoods n, as given in Table 1.

The proposed protocol collects the necessary data regarding device identities, and authenticities and stores them on the nearest edge devices. Edge devices also cooperate to transfer the data toward the sink node. Whenever any device $dev_{i}$ has data to transmit, it retrieves neighboring $N_{i}$ data from its forwarding table that fall in its transmission radius, as defined in Equation (1).

(1) $$N(dist)\;\leq \;R(dev_{i})$$

First, the source node generates route request RREQ and chooses random routes $r_{i}$ for the available list $r_{n}$, as defined in Equation (2).

(2) $$r_{n}=n_{1},\;n_{2},\;\ldots .\;,\;n_{j}$$

Later, it chooses the optimal route from $r_{i}^{\prime }$ using *K-NN* regression, a machine-learning technique [37,38]. In the proposed protocol, we set a threshold to identify and refine the selection process. Accordingly, any node $n_{j}$ that satisfies the threshold limit is given privileges for competitive computing by using a weighted function. The intelligent approach is used to compute the weighted function. For learning practice, this process is repeated, so the system can become more reliable. Three groups $G_{i}\;$, named normal, average, and weak, are made and maintained by the proposed protocol. Furthermore, each node can belong to only one group at a time, as specified in Equation (3).

(3) $$n_{i}\;\varepsilon \;G_{i}$$

The proposed protocol determines the category of the group by exploring the fitness function $f\left(n_{i}\right)$, as given in Equation (4).

(4) $$G_{i}=f\left(n_{i}\right)$$

To identify the nearest neighbors, the proposed protocol explores the *K-NN* regression machine-learning approach. First, it takes *K* closest neighbors $z_{k}$ from the source node $y_{n}$ based on Euclidean distance, as defined in Equation (5).

(5) $${\left(y_{n}\right)}_{n=1}^{k}=z_{1},\;z_{2},\;z_{3},\;\ldots ..,\;z_{k}\;$$

After determining the number of neighbors from the source node, the proposed protocol performs the fitness function for the selection of the most optimal node $x_{i}$ for data transmission, as stated in Equation (6). Unlike most of the existing approaches, the proposed protocol explores energy $e_{i}$, link estimator $l_{i}$, and packet success rate $p_{i}$ parameters. To evenly distribute the overhead and traffic distribution across the grid network, such parameters are estimated using weights α, β, and γ with uniform values.

(6) $$x_{i}=\alpha e_{i}+\beta l_{i}+\;\gamma p_{i}$$

Each node computes the value of link estimation by determining the receiving time of acknowledgements for its transmitted messages. To further optimize the selection process, the proposed protocol utilizes $f(X_{i}^{\prime }$) the n set of $x_{i}$ computation with the incorporation of error rate µ, as defined in Equation (7).

(7) $$f(X_{i}^{\prime })=\sum_{i=0}^{n}X_{i}+µ$$

**Figure 2:**
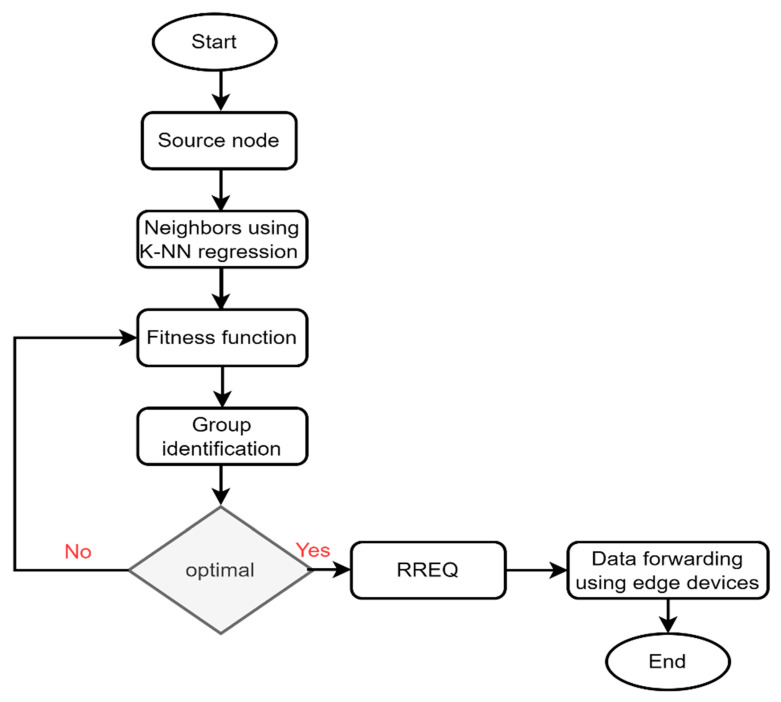
Flowchart of the proposed protocol for routing using group identification.

The edge device stores the data it received and the node’s characteristics in its database. Later, it communicates the reselection process for the forwarder node with the source node whenever any attribute value falls below than threshold. Table 2 provides the table’s format. Figure 2 shows the flowchart of the proposed protocol with sensor technologies. Its main steps are identifying the nearest nodes using the *KNN* regression and reducing the transmission cost while forwarding the data from the grid system. The group identification divides the nodes into various categories by exploring their behavior and conditions. Lastly, the optimal node is responsible for forwarding the data toward the sink node.

### 4.3. Secure Sink Coordination for Route Maintenance

The edge device verifies the data with its store data after receiving them from the border node. If the incoming request is recognized as abnormal, then the identity of the node is blocked, and information is recorded in the database. Additionally, the edge device broadcasts an alarm message to all effective route nodes. Another major contribution of the proposed protocol is to offer a secure route with the highest degree of trust. For this, the proposed protocol utilizes metrics with the integration of secret keys, hashes, and authentication parameters. All these requirements are also satisfied within the sensing nodes and edges to the sink node. We considered the sink node to be more trustworthy and to have more sufficient computational capacity than edges and sensors do, and the proposed protocol gives the sink node the primary responsibility for achieving security. First, it generates a master key $m_{k}$ and encrypts the session key $s_{k}$ for each node with associated public key $u_{i}$, as defined in Equation (8). Sink node SN is responsible for creating the key pool and its management.

(8) $$SN:\;n_{i}=Em_{k}\left(Eu_{i}\left(s_{k}\right)\right)$$

**Figure 3:**
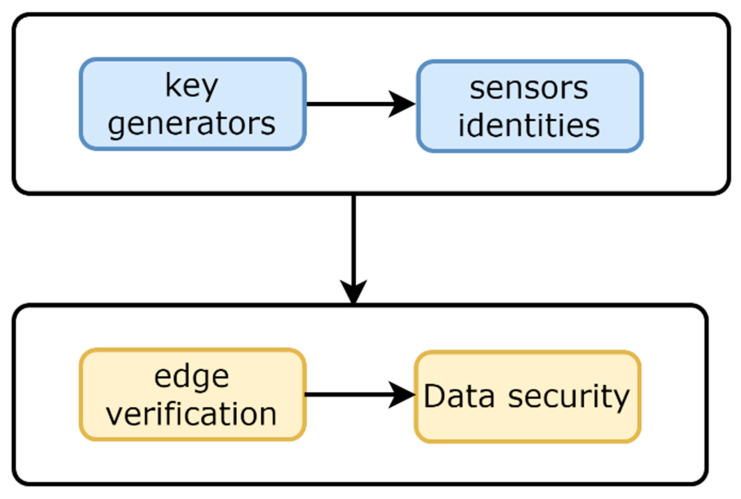
Flow diagram for security measures in the proposed protocol.

Upon receiving the key, the source node decrypts the incoming message with the $m_{k}$ of the sink node; later, it decrypts its $s_{k}$ key using a private key. In this way, each node received its key authentically and securely. Such key information is also shared by the sink node with edge devices to verify the incoming data. Each device uses blocks of data and encrypts them with their keys using cryptographic methods. Additionally, hashing methods are integrated with the encrypted data to provide integrity for sensor data in the context of a smart grid. Figure 3 depicts the procedural flow of the proposed protocol for attaining a secure edge-oriented communication system with the support of a sink node. It has two main components. One is for key generation and node authentication, while the second component is edge verification with secure data transmission. Algorithm 1 shows the steps that govern the development of the proposed protocol.
**Algorithm 1:** Secure edge-based energy management protocol**Input:**N: nodes.$s_{k}$: secret keys.
$X_{i}$: fitness function.SN: sink node.GW: gateway devices.List_N: list of neighbors.
**Output:** Data forwarders, multiple routes, nearest neighbors, authentic nodes, privacy**Procedure** multipaths  **for** (i=1; i<=N; i++)
  do   construct routing tables
   store the information** end for** **for** (i=1; i<=List_N; i++)  extract node information
id$,\;d_{i\;}$, positioning coordinates  compute fitness function $X_{i}$$=\alpha e_{i}$$+\beta l_{i}$ + γ$p_{i}$
  identify group $G_{i}$ using fitness function  compute error rate $f(X_{i}\prime$$)=\overset{n}{\underset{i=0}{\sum }}X_{i}$+ µ   **end for**
**end procedure****Procedure** authentic_comm  **for each**
$node_{i}$∈$\;[1:\;\mathrm{list}\_N_{\;}$]  **do**
  SN shares the keys for nodes and edges   Validate the incoming keys and store them in the table   **if** key= valid    call encryption ( ) **    else**     record the information in the table    **end if****end for****Procedure** data_verification( )    negotiate edge devices and SN    if authentication is verified    call data transmission ( ) **end procedure**


## 5. Simulations and Discussion

This section presents the experimental results and the system configuration. Using NS-3, the performance of the proposed protocol was evaluated in terms of varying nodes and varying mobility speeds. The sensor nodes were deployed in a grid over a 1000 × 1000 m area. With a 5 m transmission distance, the sensor nodes were positioned in the targeted environment. During the simulations, the range of sensors was from 100 to 500. The sink node was more capable in terms of computing and storage. To assess the security measures, 20 malicious nodes were deployed with 10 edge nodes. The size of the packet was 512 bits. In comparison to other work, the proposed protocol was evaluated in terms of network throughput, packet drop ratio, data latency, and energy consumption. During experimental analysis, 20 simulations were run to validate the performance of the proposed protocol and existing work. Initially, 12 simulation data were recorded in separate files to create a log for training and testing the performance results. Each simulation was run for 5000 s. Table 3 shows the list of simulation parameters with their values.

**Figure 4:**
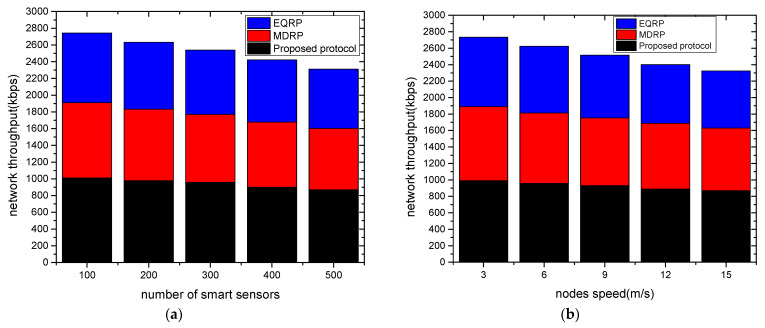
Varying smart sensors and node speed levels in terms of network throughput. (**a**) Network throughput and varying sensors; (**b**) network throughput and varying node speed.

**Figure 5:**
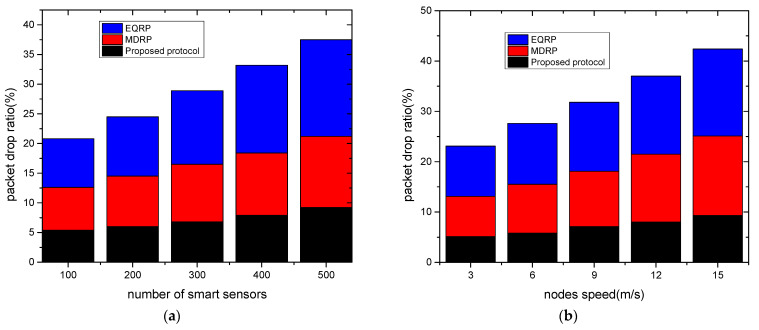
Varying smart sensors and nodes speed in terms of packet drop ratio. (**a**) Packet drop ratio and varying sensors; (**b**) packet drop ratio and varying node speed.

**Figure 6:**
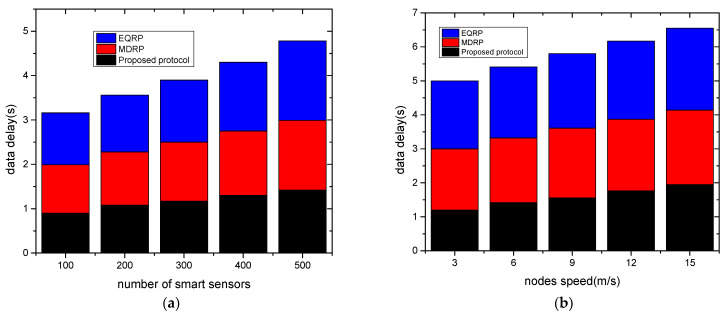
Varying smart sensors and nodes speed in terms of data delay. (**a**) Data delay and varying sensors; (**b**) data delay and varying node speed.

**Figure 7:**
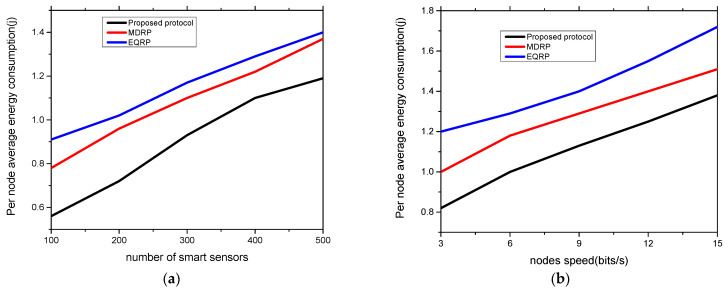
Varying smart sensors and nodes speed in terms of energy consumption. (**a**) Energy consumption and varying sensors; (**b**) energy consumption and varying node speed.

The experimental findings for network throughput against two scenarios are shown in Figure 4a,b. Under a varying number of devices and varying node speed, the proposed protocol gave a higher network throughput by averages of 15% and 18% as compared to existing work. This was due to the multi-hop transmission of data from the sensors to the edge devices, and from the edges to the cloud systems. The proposed protocol also used correlation analysis to select the most trustworthy neighbors from the routing tables, and it recorded the updated data for preparing the system for a timely response. Furthermore, the security of the proposed protocol with the coordination of the sink node lessens the possibility of unwanted data traffic that interferes with the transmission system. Figure 5a,b show the evaluation of the proposed protocol and related work in terms of packet loss rates under a varying number of sensors and varying speed rates. Based on the findings, the proposed protocol significantly lowered the ratio of packet drops by an average of 10% and 13% when compared to other work. This is because, when they reached their threshold limits, intelligent and secure routes were obtained based on updated policies. Furthermore, to train the system and store the estimated weight value in the forwarding tables, the fitness function uses a variety of realistic characteristics. These values assist in balancing the data traffic over the communication. Figure 6a,b show the experimental results for the proposed protocol and existing work using various sensors and mobility speed levels. According to the findings, the proposed methodology greatly reduced the data delay by an average of 12% and 15% compared to other relevant studies. This is because of the selection of data forwarders using correlation analysis and predicting the most optimal value for the transmission of grid data while reducing unnecessary processing delays. The proposed protocol also included edges between sensors and sink nodes, which reduced the response time for the requested devices and efficiently managed the links for forwarding the data of smart sensors. Moreover, the communication sessions were kept secure and validated periodically to increase the longevity of the selected paths. Such methods in the proposed protocol reduce the possibility of communication failure and additional computing demands. Under varying sensors and mobility speed levels, the efficiency of the proposed protocol was evaluated for energy consumption, as shown in Figure 7a,b. According to the analysis of the results, the proposed protocol reduced energy consumption by an average of 11% and 14% when compared to other studies. This was a result of the introduction of reasonable multi-paths that balanced the distribution of energy resources across the devices using an intelligent approach. Furthermore, the proposed protocol used KNN regression analysis to update the route information in the neighboring tables without excessive complexity for the smart grid system. Unlike most of the existing work, the proposed protocol secures the communication routes by integrating authentic sessions among devices, edges, and remote systems. Such an approach reduces the control overheads on the nodes and ultimately increases the energy efficiency of the grid systems.

## 6. Conclusions

WSN and IoT are integrated with various industrial applications for the smart monitoring of communication systems. This not only provides remote data collection but also increases the efficacy of wireless devices. In a smart grid, smart sensors and IoT devices perform crucial functions for coping with data gathering and forwarding practices. However, most proposed methods provide energy-efficient solutions at the cost of a huge number of data retransmissions and are not able to balance the overhead in the case of node mobility. In addition, many solutions fail to respond to the query of the requested device due to a lack of optimal routes. Ineffective decisions result in increased energy usage for smart grid applications and a shorter network lifetime. In this study, the following contributions were achieved.

- A secure edge-based sensing protocol was proposed that uses correlation analysis and node behavior based on performance parameters.
- The presence of edges provides prompt responses to the system in crucial circumstances.
- Even in the presence of network threats, sink-oriented collaborative security raises the level of trust across communication systems.

In future work, to increase the scalability and further reduce the overheads on smart sensors, we intend to combine the architecture of a software-defined network with the proposed protocol.

## Figures and Tables

**Table 1:** Format of the forwarding table.

1 Byte	1 Byte	1 Byte	1 Byte	2 Bytes
Identity id	Distance $d_{i\;}$	positioning coordinates x,y	neighborhoods n	fitness $f\left(x\right)$

**Table 2:** Format of the edge table.

Identity	Fitness Vlue	Group	Attributes		
ID	$f\left(x\right)$	$G_{i}$	$e_{i}$	$l_{i}$	$p_{i}$

**Table 3:** List of simulation parameters.

Parameters	Values
Simulation area	1000 m × 1000 m
Sensor nodes	100–500
Mobility pattern	Random
Node mobility	3 m/s to 15 m/s
Malicious nodes	20
Energy of nodes	5J
Packet size	512 bits
Number of sink nodes	1
Control message	25 bits
Transmission distance	5 m
Traffic type	CBR
Individual simulation time	5000 s
Edge nodes	10

## Data Availability

All data are available in the manuscript.
